# Determinants of facility delivery after implementation of safer mother programme in Nepal: a prospective cohort study

**DOI:** 10.1186/1471-2393-13-193

**Published:** 2013-10-20

**Authors:** Rajendra Karkee, Colin W Binns, Andy H Lee

**Affiliations:** 1 School of Public Health and Community Medicine, BP Koirala Institute of Health Sciences, Dharan, Nepal; 2 School of Public Health, Curtin University, Perth, WA, Australia

**Keywords:** Determinants, Factors, Facility delivery, Childbirth, Maternal health services, Nepal

## Abstract

**Background:**

There are several barriers for pregnant women to deliver in a health care facility. This prospective cohort study investigated factors affecting facility delivery and reasons for unplanned place of delivery after implementation of the safer mother programme in Nepal.

**Methods:**

Baseline interviews using a validated questionnaire were conducted on a sample of 700 pregnant women representative of the Kaski district in central Nepal. Follow-up interviews of the cohort were then conducted within 45 days postpartum. Stepwise logistic regression analysis was performed to determine factors associated with the facility delivery outcome.

**Results:**

Of the 644 pregnant women whose delivery location had been identified, 547 (85%) gave birth in a health care facility. Women were more likely to deliver in a health facility if they were educated especially with higher secondary or above qualification (adjusted odds ratio (OR) 12.39, 95% confidence interval (CI) 5.09 to 30.17), attended 4 or more antenatal care visits (OR 2.15, 95% CI 1.25 to 3.69), and lived within 30 minutes to the facility (OR 11.61, 95% CI 5.77 to 24.04). For the 97 women who delivered at home, 72 (74.2%) were unplanned, mainly due to quick precipitation of labour making it impossible to reach a health facility.

**Conclusions:**

It appeared that facility delivery occurs more frequent among educated women and those who live nearby, even though maternity services are now freely available in Nepal. Because of the difficult terrain and transportation problem in rural areas, interventions that make maternity service physically accessible during antenatal period are needed to increase the utilisation of health facility for child birth.

## Background

Almost all maternal deaths occur in developing countries [1], where the majority of women deliver at home without skilled birth attendance. These mothers are at increased risk from unpredictable obstetric complications, often ending in death either at home or after transfer to a health facility [2,3]. Many of these deaths could be avoided by the provision of skilled care, preferably at a health care facility [4]. Consequently, the safe motherhood programme aims to increase the proportion of facility delivery by identifying and overcoming barriers in the utilisation of delivery service by pregnant women.

In Nepal, the government has promoted several interventions including the establishment of maternity waiting homes [5], birth preparedness and complication readiness, as well as a safer mother programme to increase the proportion of delivery at health care facility [6]. The safer mother programme consists of: (1) a safe delivery incentive component (initiated in July 2005), that provides cash incentives to women who deliver in a designated health facility; and (2) provision of free delivery care for normal, complicated, and caesarean section births (initiated in January 2009) [7]. In spite of these efforts, 65% of women still deliver at home [8], which presents a challenge for Nepal in reducing maternal mortality to achieve the Millennium Development Goal 5.

Barriers in accessing adequate obstetric care may be classified into three delays [3,9]. The 'first delay’ is related to decision to seek care; the 'second delay’ factors are causes of delay in travelling to the facility; whereas the 'third delay’ concerns access to care after arrival at the health facility. Twenty factors associated with the first two phases of delays have been identified as potential barriers to delivery service utilisation, but they remain inconsistent between studies [9]. This implies that determinants are context specific and their effects vary from one geographic and social setting to another. Previous studies in Nepal were either analyses of the Demographic Health Survey [10-13] or cross sectional studies [14-16], conducted before implementation of the safer mother programme.

In the present prospective cohort study, we investigate factors affecting facility delivery and reasons for unplanned place of delivery after implementation of the safer mother programme in Nepal.

## Methods

### Study setting and location

This study was conducted in the Kaski district of the Western Development Region of Nepal, a hilly area with a population of 455,000 in 2010 [17]. The district is administratively divided into 42 Village Development Committees (VDC) and two municipalities. Geographically, the district has a central valley housing the two municipalities and a few VDCs in the urban areas. The rest of the VDCs spread out into the rural hilly terraces. Pokhara is the regional capital which attracts people from neighbouring districts for education, employment and health care. It has a regional government hospital, two teaching hospitals of private medical colleges and several nursing homes and pharmacies. The three hospitals provide free delivery services and serve as referral centres for emergency obstetric care. The majority of deliveries took place in the public hospital (80%), followed by private hospitals (16%) and birth centres (4%). In the year July 2011 to August 2012, the public hospital received about 7500 delivery cases, of which 350 (4.6%) involved complications and 1700 (22.6%) underwent caesarean section [18].

For the purpose of health service provision, the District Public Health Office of Kaski has divided the rural district into *illakas*. Each *illaka* includes several VDCs and at least one functioning birth centre to provide free basic emergency obstetric care services. Monetary incentives are provided to women who attend the recommended four antenatal care visits and deliver at a birth centre. Rural areas are connected with the central urban valley by non-gravelled roads, but transportation is infrequent and often obstructed during the monsoon season.

The government of Nepal has classified the population into upper caste, lower caste, janajati and religious minorities. 'Upper caste’ and 'lower caste’ people have Indo-Aryan origin whereas 'janajati’ refers to the Tibeto-Burman people. In the Kaski district, about half (49%) of the population belong to upper caste, 34% belong to janajati, while 16% are classified as lower caste and the remaining 1% as religious minorities. The female population in the 15–49 age group is 117,500 with 13,800 expected pregnancies annually [17]. Our study population consisted of all pregnant female residents who delivered during the period December 2011 to October 2012.

### Study design and participants

A community-based prospective cohort study was conducted to recruit women who were 5 months or more pregnant from the two urban municipalities. For rural areas 7 of the 13 *illakas* were chosen at random. Within each selected *illaka*, a village that contained the birth facility was purposely chosen. All pregnant women in that village were invited to participate. Additional women were recruited from another adjacent village whenever necessary. Of the total 748 pregnant women (398 from urban, 350 from rural) approached, 701 (380 from urban, and 321 from rural) agreed to participate in the baseline survey, giving an overall response rate of 93.7%.

### Data collection and ethics

The study questionnaire was taken from the National Health and Demographic Survey, an instrument that has been widely used and validated in many countries, including Nepal [8]. The translated questionnaire was pretested on 50 pregnant women for cultural appropriateness, content validity and understanding. Baseline interviews, conducted between December 2011 and January 2012, sought information on socio-demographic and household characteristics including assets, childbirth history, knowledge and preparedness about pregnancy and child birth, and time to reach the nearest birth centre in rural areas and hospital in urban areas. A follow-up second interview was conducted within 45 days postpartum to obtain information on utilisation of antenatal and delivery care service.

From the selected areas, 15 female local residents were recruited and employed as data enumerators. All of them had completed 10th grade education and possessed basic knowledge on maternity issues. They were responsible for searching and identifying pregnant women in their localities, and trained by the first author to conduct the subsequent face-to-face interviews. The project protocol was approved by the Human Research Ethics Committee of Curtin University (approval number HR 130/2011), Ethical Review Board of Nepal Health Research Council (approval number 88/2011) and the District Public Healcprth Office of Kaski. An information sheet was provided and read to each participant before obtaining her signed or thumb-print informed consent. Confidentiality of the information provided was maintained throughout the study. Participants were assured of their freedom to withdraw without any negative consequences.

### Statistical analysis

The main socio-demographic and maternity related variables are listed in Table 1. An asset score for socioeconomic status was generated from the first component of a principal components analysis [19], utilising survey questions related to household assets. The asset score was then used to develop wealth quintiles. Distance to the nearest birth facility was estimated by the time took (either on foot or by vehicle) to reach the facility and categorised as less than 30 min, between 30 and 60 min, and over 60 min. Four levels of education level was recorded: none, primary (1-5th grade), secondary (6-10th grade), higher secondary or above (after 10th grade). Employment status was categorised as employed (full-time salaried job), semi-employed (wage based labour, small business or employed aboard), and unemployed (agricultural, housewife, or jobless). For caste, only 3 respondents belonged to religious minorities and they were merged with the janajati group to facilitate analysis.

**Table 1:** Characteristics of participants (n = 644) by delivery location, Kaski, Nepal, 2012

**Characteristics**	**Home delivery**	**Facility delivery**	**Total**	**Chi-square test p-value**
n	97	547	644	
Women’s age (years)				0.640
15-19	17 (17.5%)	76 (13.9%)	93 (14.4%)	
20-24	47 (48.5%)	279 (51%)	326 (50.6%)	
25-40	33 (34%)	192 (35.1%)	225 (34.9%)	
Parity				0.208
0	42 (43.3%)	292 (53.4%)	334 (51.9%)	
1	30 (30.9%)	149 (27.2%)	179 (27.8%)	
2	15 (15.5%)	73 (13.3%)	88 (13.7%)	
3-7	10 (10.3%)	33 (6%)	43 (6.7%)	
Women’s education level				< 0.001
No education	24 (24.7%)	31 (5.7%)	55 (8.5%)	
Primary	27 (27.8%)	107 (19.6%)	134 (20.8%)	
Secondary	34 (35.1%)	204 (37.3%)	238 (37%)	
Higher secondary or above	12 (12.4%)	205 (37.5%)	217 (33.7%)	
Husband’s education level				< 0.001
No education	4 (4.1%)	6 (1.1%)	10 (1.6%)	
Primary	32 (33%)	93 (17%)	125 (19.4%)	
Secondary	44 (45.4%)	204 (37.3%)	248 (38.5%)	
Higher secondary or above	17 (17.5%)	244 (44.6%)	261 (40.5%)	
Husband’s employment				0.003
Employed	7 (7.2%)	105 (19.2%)	112 (17.4%)	
Semi-employed	67 (69.1%)	363 (66.4%)	430 (66.8%)	
Unemployed	23 (23.7%)	79 (14.4%)	102 (15.8%)	
Residential location				< 0.001
Rural	64 (66%)	233 (42.6%)	297 (46.1%)	
Urban	33 (34%)	314 (57.4%)	347 (53.9%)	
Caste				< 0.001
Lower caste	42 (43.3%)	119 (21.8%)	161 (25.1%)	
Janajati	15 (15.5%)	125 (22.9%)	140 (21.8%)	
Upper Caste	40 (41.2%)	301 (55.2%)	341 (53.1%)	
Household wealth quintile				< 0.001
1 (lowest)	39 (41.1%)	99 (18.2%)	138 (21.6%)	
2	25 (26.3%)	97 (17.8%)	122 (19.1%)	
3	14 (14.7%)	107 (19.6%)	121 (18.9%)	
4	10 (10.5%)	114 (20.9%)	124 (19.4%)	
5 (highest)	7 (7.4%)	128 (23.5%)	135 (21.1%)	
Information received on pregnancy and delivery				0.790
No	23 (23.7%)	123 (22.5%)	146 (22.7%)	
Yes	74 (76.3%)	424 (77.5%)	498 (77.3%)	
Maternity issues discussed with family				0.014
No	47 (50%)	200 (36.6%)	247 (38.6%)	
Yes	47 (50%)	346 (63.4%)	393 (61.4%)	
Frequency of antenatal care visit				0.009
< 4	35 (37.2%)	133 (24.3%)	168 (26.2%)	
≥ 4	59 (62.8%)	414 (75.7%)	473 (73.8%)	
Distance to nearest health facility				< 0.001
≤ 30 minutes	22 (22.7%)	355 (64.9%)	377 (58.5%)	
31–60 minutes	46 (47.4%)	144 (26.3%)	190 (29.5%)	
> 60 minutes	29 (29.9%)	48 (8.8%)	77 (12%)	

The outcome variable was place of delivery. Univariate statistics were first conducted to compare the profile of participants between the home delivery and facility delivery groups. Stepwise logistic regression analysis was then performed to ascertain the determinants of facility delivery. Independent variables considered in the regression model were either significant factors from the univariate analysis or plausible confounders from previous studies in Nepal [11,15,16]. Crude and adjusted odds ratios (OR) and corresponding 95% confidence intervals (CI) for the facility delivery prevalence were reported. All statistical analyses were performed in the SPSS package version 18 [20].

## Results

### Participant characteristics

The average gestational age of women at recruitment was 27.9 weeks (range 16 to 38 weeks). Majority of the participants were under 25 years of age (62%), received primary or above education (92%), and were unemployed (79%). More than half of them were expecting their first child (52%), living with their extended family (64%), and belonged to the upper caste (52%). Of the 338 women who had given birth before, 62% had used a health facility and 21% had some complications for the last delivery. Almost all (98%) pregnant women had made at least one antenatal care visit. On average, it took 62 (SD 39) min for rural women travelled to the nearest birth centre but only 31(SD 15) min for urban women.

### Facility delivery

Of the 701 baseline respondents, place of delivery was identified for 644 (92%) women from their second interview. However, 43 women were lost to follow up, 9 had a prenatal death, and 5 delivered on their way to a facility. About 90% of women from urban area and 78% of women from rural area delivered in a health care facility. Among the 547 facility deliveries, 77 (14%), 419 (76.6%) and 51 (9.3%) women delivered at birth centres, regional public hospital and private hospitals, respectively. About 48% of women delivered within 5 hours of reaching a health care facility and 90% delivered within 25 hours of arrival.

### Comparison between home delivery and facility delivery groups

Table 1 presents the characteristics of the participants by place of delivery. The two groups appeared to be different in terms of caste, household wealth, education level (both women and their husband), husband’s employment status, residential location, and distance to the nearest health facility. Moreover, a higher proportion of women in the facility delivery group attended four or more antenatal care visits and discussed maternity issues with family members than their counterparts who delivered at home.

Table 2 shows the results of logistic regression analysis. Education level, distance to the nearest health facility, and frequency of antenatal care visits emerged as significant determinants of facility delivery, after accounting for the apparent collinearity between plausible confounding factors. Women who completed the recommended four antenatal visits were more likely to deliver at a facility than those without (adjusted OR 2.15, 95% CI 1.25 to 3.69). Similarly, women tended to deliver at a health facility if they were educated especially with higher secondary or above qualification (adjusted OR 12.39, 95% CI 5.09 to 30.17), or lived within 30 minutes to the nearest facility (adjusted OR 11.61, 95% CI 5.77 to 24.04).

**Table 2:** Determinants of facility delivery from backward stepwise logistic regression, Kaski, Nepal, 2012

	**Crude OR (95% CI)**	**Adjusted OR (95% CI)**	**p value**
Women’s education level			< 0.001
No education	1	1	
Primary	3.07 (1.55, 6.05)	3.57 (1.60, 7.94)	
Secondary	4.64 (2.43, 8.85)	5.66 (2.62, 12.22)	
Higher secondary or above	13.22 (6.0, 29.11)	12.39 (5.09, 30.17)	
Frequency of antenatal care visit			0.005
< 4	1	1	
≥ 4	1.84 (1.16, 2.93)	2.15 (1.25, 3.69)	
Distance to nearest health facility			< 0.001
≤ 30 minutes	9.74 (5.18, 18.32)	11.61 (5.77, 24.04)	
31–60 minutes	1.89 (1.07, 3.33)	1.72 (0.93, 3.19)	
> 60 minutes	1	1	

### Reasons for unplanned home and facility delivery

Within the facility delivery group, 75 (13.7%) women said they had not initially planned to deliver at a facility. Table 3 lists the most popular reasons given for their unplanned delivery. As expected, almost half of them reported development of complications at home or following the advice of a health worker. For the 97 women who delivered at home, 72 (74.2%) were unplanned, mainly due to quick precipitation of labour making it impossible to reach a health facility.

**Table 3:** Most important reasons for unplanned delivery, Kaski, Nepal, 2012

**Unplanned facility delivery (n = 75)**		**Unplanned home delivery (n = 72)**	
**Reasons**	**Frequency**	**Reasons**	**Frequency**
Developed complications at home	32 (42.7%)	Labour too quick to reach health facility	53 (73.6%)
On health worker’s advice	33 (44%)	Lack of transport or nearby facility	8 (11.1%)
On friends or neighbours’ advice	10 (13.3%)	No one to accompany to facility	4 (5.6%)
		Family custom	7 (9.7%)

## Discussion

The observed facility delivery prevalence of 85% is comparable to the estimate of 81% in 2010 provided by the District Public Health Office of Kaski [17], but much higher than the 35% facility delivery reported by the National Demographic Health Survey [8]. A similar rate of facility delivery (81%) was obtained in previous studies conducted in the urban Kathmandu valley [16] and Pokhara city [21]. Moreover, health system surveillance data revealed an average 43% facility delivery rate with a wide range (7% to 93%) across districts [6]. Such geographical differences in utilisation should be taken into account when planning for service expansion and allocation of resources by the government.

Women’s education level, distance to the nearest health facility and frequency of antenatal care visits were found to be significantly associated with the likelihood of facility delivery. Our results are consistent with the literature [9,22]. Previous studies in Nepal had also shown that a distance of more than one hour to the facility could exert a negative impact on delivery service use [11], while mothers with no education were more likely to deliver at home [15].

Birth preparedness was high in the study district and almost all women made at least one antenatal visits. Health workers at the birth centres and hospitals counselled women about preparation activities and danger signs of pregnancy and delivery. Female community health volunteers provided information to pregnant women in the communities and encouraged them to use the nearest health facility for delivery [23].

Apparently, demographic factors such as age, parity, household wealth and caste became non-significant in the stepwise regression model, even though they appeared to be plausible predictors of facility delivery in the univariate analysis as well as previous studies in Nepal [15,16] or other countries [24-29]. Firstly, these factors are highly correlated with the women’s education level. Secondly, the discrepancy in results might be due to the introduction of incentive scheme and the provision of free delivery care after implementation of the safer mother programme. In the past few years, maternity services have reached out to different social strata with regards to age, wealth and caste. Similar effects have been reported by studies in Burkina Faso [30] and Ghana [31] after the reduction of user fees. Indeed, data from the District Public Health Office of Kaski showed that facility utilisation increased from 49% in 2008 to 72% in 2009 and 81% in 2010. Increases in facility delivery have also been observed in other districts of Nepal [7].

Because of the hilly landscape, poor or non-existent roads and the absence of systematic transport in rural areas, distance remains as the major obstacle to use a health facility. Even in urban areas, the distance and availability of transport affect the timely utilisation of delivery services. The main reason behind unplanned home birth was quick precipitation of labour, followed by lack of transport or nearby facility, similar to other studies in Nepal [16,21] and India [27].

There are several options to be considered to further increase the rate of facility delivery. One possibility is to establish more birth centres. However, building new or upgrading existing facilities that can provide comprehensive emergency obstetric care within easy reach of every rural woman is unlikely to be a feasible solution for Nepal in the foreseeable future. An arrangement for rapid transportation in rural areas, which help women to reach a rural birth centre or a nearby hospital quickly, also poses as a challenging logistic problem due to the difficult terrain and resource constraints.

Maternity waiting homes at or near the health facility may offer a viable option particularly for women residing in remote areas, whereby they could arrive early before the due date and wait for their delivery. Maternity waiting homes have been used in other countries but they generally differ in structure and provision of services, resulting in varying degrees of success [32]. Although maternity waiting homes had been constructed and introduced in rural west Nepal, they were not utilised effectively by pregnant women, possibly due to unawareness of their availability and/or lack of adequate facilities [5]. Improving and increasing maternity waiting homes may be an acceptable and affordable way to enhance the facility delivery rate and should be further investigated.

Several issues should be considered when interpreting the findings. The present study was conducted on mostly literate, young, nulliparous women, in a district of Nepal which has a relatively high adult literacy rate of 66.8% and ranks third in terms of the human development index amongst the 75 districts in Nepal [33]. This might limit the generalizability of our findings to the whole country. Selection bias could not be ruled out because all participants were voluntary. Our recruitment process, nevertheless, ensured that they were representative of the pregnant women population in the entire catchment region of Kaski district. Moreover, to improve the accuracy of their responses, face-to-face interviews were conducted by female data enumerators who were residents of the selected areas and thus aware of the local context and issues.

## Conclusions

In this study, women’s education level, frequency of antenatal care visits, and distance to the nearest health facility were found to be significant determinants of facility delivery, even though maternity services are now freely available in Nepal, suggesting that such services are more likely to be utilised when they are nearby. Because of the difficult terrain and transportation problem in rural areas, interventions that make maternity service physically accessible during antenatal period are needed to increase the utilisation of health facility for child birth. In particular, the option of offering maternity waiting homes for women close to delivery should be considered for further investigation.

## Competing interests

The authors declare that they have no competing interest.

## Authors’ contributions

RK managed the project and data collection, performed statistical analysis and drafted the manuscript. CWB developed the study protocol and revised the manuscript. AHL contributed to the study design, data analysis and revision of the manuscript. All three authors read and approved the final version for publication. All authors read and approved the final manuscript.

## Pre-publication history

The pre-publication history for this paper can be accessed here:

http://www.biomedcentral.com/1471-2393/13/193/prepub

## References

[B1] Hill K Thomas K AbouZahr C Walker N Say L Inoue M Suzuki E Estimates of maternal mortality worldwide between 1990 and 2005: an assessment of available data The Lancet 2007 370 9595 1311 1319 10.1016/S0140-6736(07)61572-4 17933645

[B2] Ronsmans C Graham WJ Maternal mortality: who, when, where, and why The Lancet 2006 368 9542 1189 1200 10.1016/S0140-6736(06)69380-X 17011946

[B3] Thaddeus S Maine D Too far to walk: maternal mortality in context Soc Sci Med 1994 38 8 1091 1110 10.1016/0277-9536(94)90226-7 8042057

[B4] Filippi V Ronsmans C Campbell OMR Graham WJ Mills A Borghi J Koblinsky M Osrin D Maternal health in poor countries: the broader context and a call for action The Lancet 2006 368 9546 1535 1541 10.1016/S0140-6736(06)69384-7 17071287

[B5] Shrestha D Rajendra P Shrestha N Feasibility study on establishing maternity waiting homes in remote areas of Nepal Region Health Forum 2007 11 2 33

[B6] Ministry of Health and Population (MoHP) Annual report of department of health services (2010/2011) 2011 Kathmandu: Government of Nepal, Ministry of Health and Population

[B7] Witter S Khadka S Nath H Tiwari S The national free delivery policy in Nepal: early evidence of its effects on health facilities Health Policy Plan 2011 26 suppl 2 ii84 ii91 22027923 10.1093/heapol/czr066

[B8] Ministry of Health and Population (MoHP) [Nepal], New ERA, ICF International Inc Nepal demographic and health survey 2011 2012 Kathmandu: Government of Nepal, Ministry of Health and Population

[B9] Gabrysch S Campbell OMR Still too far to walk: literature review of the determinants of delivery service use BMC Pregnancy Childbirth 2009 9 34 10.1186/1471-2393-9-34 19671156 PMC2744662

[B10] Furuta M Salway S Women’s Position within the household as a determinant of maternal health care use in Nepal Int Fam Plan Perspect 2006 32 1 17 27 10.1363/3201706 16723298

[B11] Hotchkiss DR Expansion of rural health care and the use of maternal services in Nepal Health Place 2001 7 39 45 10.1016/S1353-8292(00)00036-8 11165154

[B12] Matsumura M Gubhaju B Women’s status household structure and the utilisation of maternal health services in Nepal Asia-Pacific Popul J 2001 2 52 54

[B13] Sharma SK Sawaingdee Y Sirirassamee B Access to health: Women’s status and utilization of maternal health services in Nepal J Biosoc Sci 2007 39 05 671 692 10.1017/S0021932007001952 17359562

[B14] Thapa N Chongsuvivatwong V Geater AF Ulstein M High-risk childbirth practices in remote Nepal and their determinants Women Health 2001 31 4 83 97 10.1300/J013v31n04_06 11310813

[B15] Wagle R Sabroe S Nielsen B Socioeconomic and physical distance to the maternity hospital as predictors for place of delivery: an observation study from Nepal BMC Pregnancy Childbirth 2004 4 1 8 10.1186/1471-2393-4-8 15154970 PMC425583

[B16] Bolam A Manandhar DS Shrestha P Ellis M Malla K Costello AM Factors affecting home delivery in the Kathmandu valley, Nepal Health Policy Plan 1998 13 2 152 158 10.1093/heapol/13.2.152 10180403

[B17] District Public Health Office Kaski Annual health report of kaski 2012 GoN, Pokhara: Regional Health directorate, Ministry of Health and Population

[B18] Family Health Division/ Nepal Health Sector Support Programme Responding to increased demand for institutional childbirths at referral hospitals in Nepal: situational analysis and emerging options 2013 Kathmandu: Ministry of Health and Population

[B19] Filmer D Pritchett LH Estimating wealth effects without expenditure data-or tears: an application to educational enrollments in states of India Demography 2001 38 1 115 132 11227840 10.1353/dem.2001.0003

[B20] IBM SPSS PASW statistics for window version18 2010 Chicago: SPSS Inc

[B21] Sreeramareddy CT Joshi HS Sreekumaran BV Giri S Chuni N Home delivery and newborn care practices among urban women in western Nepal: a questionnaire survey BMC Pregnancy Childbirth 2006 6 27 10.1186/1471-2393-6-27 16928269 PMC1560161

[B22] Karkee R Lee AH Binns CW Why women don't utilise maternity services in Nepal: a literature review WHO South-East Asia Journal of Public Health 2013 in press 10.4103/2224-3151.206759 28615588

[B23] Karkee R Lee AH Binns CW Birth preparedness and skilled attendance at birth in Nepal: implications for achieving millennium development goal 5 Midwifery 2013 http://dx.doi.org/10.1016/j.midw.2013.05.002 10.1016/j.midw.2013.05.002 23751594

[B24] Agha S Carton TW Determinants of institutional delivery in rural Jhang, Pakistan Int J Equity Health 2011 10 31 10.1186/1475-9276-10-31 21801437 PMC3159141

[B25] Babalola S Fatusi A Determinants of use of maternal health services in Nigeria - looking beyond individual and household factors BMC Pregnancy Childbirth 2009 9 1 43 10.1186/1471-2393-9-43 19754941 PMC2754433

[B26] Mayhew M Hansen PM Peters DH Edward A Singh LP Dwivedi V Mashkoor A Burnham G Determinants of skilled birth attendant utilization in Afghanistan: a cross-sectional study Am J Public Health 2008 98 10 1849 1856 10.2105/AJPH.2007.123471 18703445 PMC2636465

[B27] Das S Bapat U More N Chordhekar L Joshi W Osrin D Prospective study of determinants and costs of home births in Mumbai slums BMC Pregnancy Childbirth 2010 10 1 38 10.1186/1471-2393-10-38 20670456 PMC2928174

[B28] Duong DV Binns CW Lee AH Utilization of delivery services at the primary health care level in rural Vietnam Soc Sci Med 2004 59 2585 2595 10.1016/j.socscimed.2004.04.007 15474211

[B29] Kesterton A Cleland J Sloggett A Ronsmans C Institutional delivery in rural India: the relative importance of accessibility and economic status BMC Pregnancy Childbirth 2010 10 1 30 10.1186/1471-2393-10-30 20525393 PMC2898676

[B30] De Allegri M Ridde V Louis VR Sarker M Tiendrebeogo J Ye M Muller O Jahn A Determinants of utilisation of maternal care services after the reduction of user fees: a case study from rural Burkina Faso Health Policy 2011 99 3 210 218 10.1016/j.healthpol.2010.10.010 21056505

[B31] Mills S Williams J Adjuik M Hodgson A Use of health professionals for delivery following the availability of free obstetric care in northern Ghana Matern Child Health J 2008 12 4 509 518 10.1007/s10995-007-0288-y 17955355

[B32] Hussein J Kanguru L Astin M Munjanja S The effectiveness of emergency obstetric referral interventions in developing country settings: A systematic review PloS Med 2012 9 7 e1001264 10.1371/journal.pmed.1001264 22807658 PMC3393680

[B33] UNDP Nepal human development report 2004: empowerment and poverty reduction 2004 Kathmandu: United Nations Development Programme

